# Identification of Prognosis Biomarkers for High-Grade Serous Ovarian Cancer Based on Stemness

**DOI:** 10.3389/fgene.2022.861954

**Published:** 2022-03-14

**Authors:** Zhihang Wang, Lili Yang, Zhenyu Huang, Xuan Li, Juan Xiao, Yinwei Qu, Lan Huang, Yan Wang

**Affiliations:** ^1^ Key Laboratory of Symbolic Computation and Knowledge Engineering, Ministry of Education, College of Computer Science and Technology, Jilin University, Changchun, China; ^2^ Department of Obstetrics, The First Hospital of Jilin University, Changchun, China; ^3^ College of Artificial Intelligence, Jilin University, Changchun, China

**Keywords:** ovarian cancer, high-grade serous ovarian cancer, cancer stem cells, mRNAsi, single-cell, gene biomarkers

## Abstract

In this paper, high-grade serous ovarian cancer (HGSOC) is studied, which is the most common histological subtype of ovarian cancer. We use a new analytical procedure to combine the bulk RNA-Seq sample for ovarian cancer, mRNA expression-based stemness index (mRNAsi), and single-cell data for ovarian cancer. Through integrating bulk RNA-Seq sample of cancer samples from TCGA, UCSC Xena and single-cell RNA-Seq (scRNA-Seq) data of HGSOC from GEO, and performing a series of computational analyses on them, we identify stemness markers and survival-related markers, explore stem cell populations in ovarian cancer, and provide potential treatment recommendation. As a result, 171 key genes for capturing stem cell characteristics are screened and one vital cancer stem cell subpopulation is identified. Through further analysis of these key genes and cancer stem cell subpopulation, more critical genes can be obtained as LCP2, FCGR3A, COL1A1, COL1A2, MT-CYB, CCT5, and PAPPA, are closely associated with ovarian cancer. So these genes have the potential to be used as prognostic biomarkers for ovarian cancer.

## 1 Introduction

Currently, ovarian cancer is not a single disease and can be subdivided into at least five different histological subtypes with diverse identifiable risk factors, cellular origin, molecular compositions, clinical features, and treatment approaches (Prasetyanti and Medema, 2017). Ovarian cancer is a global problem, often diagnosed at an advanced stage, and there are currently no effective screening strategies (Matulonis et al., 2016). For ovarian cancer research, omics big data research provides a new biological perspective for ovarian cancer and offers a valuable reference for the pathophysiology and treatment strategies of ovarian cancer patients (Ince et al., 2015). Improving the genomic understanding of the histological subtypes of ovarian cancer has been an important goal for researchers. This goal can promote researchers to understand the risk factors associated with the disease and develop prevention and treatment strategies.

Because ovarian cancer has many subtypes, it leads to strong tumor heterogeneity. Tumor heterogeneity is one of the characteristics of malignant tumors, that is, tumor tissue consists of cell populations with different expression profiles or biological functions, which will lead to differences in tumor growth rate, invasion and metastasis ability, drug sensitivity, and other aspects (Kim et al., 2021). Tumor heterogeneity not only leads to tumor recurrence, metastasis, and drug resistance but also directly affects clinical treatment. An in-depth study on the formation and regulation mechanism of tumor heterogeneity will provide a theoretical basis for precisely targeted therapy of tumors. With the rapid development of single-cell sequencing technology, researchers can study the biochemical process and the pathogenesis of some diseases at the single-cell level. Single-cell sequencing technology has been widely used in tumor, developmental biology, clinical diagnosis of tumor and stem cell development and differentiation, and so on (Navin and Hicks, 2011). Tumor single-cell sequencing can be studied at the single-cell level in many ways. For example, the heterogeneous tumor, tumor microenvironment, tracking the metastasis and diffusion of cancer cells, understanding the evolution of drug resistance of cancer cells during drug therapy.

Each of these ovarian cancer cell types may represent either a hierarchy of CSC or an entirely different population of CSC for that particular ovarian histotype (Steffensen et al., 2011). Ovarian cancer stem cells have unique genetic characteristics that enable them to reproduce the ability of the original tumor to proliferate with chemotherapy and promote relapse. The molecular characteristics of these cells may explain some of the unique characteristics of CSCs that control self-renewal and metastasis (Alvero et al., 2011). In ovarian cancer, abnormal canonical and atypical WNT signaling pathways are involved in CSC survival tumor volume expansion and invasion/metastasis (Katoh, 2017). All these indicate that the heterogeneity of ovarian cancer is closely related to tumor stem cells.

There are growing interest in cancer stem cells (CSCs). CSCs can self-renew, proliferate infinitely and form heterogeneous tumor cell populations. mRNAsi can be used to evaluate stemness. Higher mRNAsi scores are associated with active biological processes in CSCs and greater tumor dedifferentiation, as reflected by histopathological grades (Vlashi and Pajonk, 2015). CSCs play a crucial role in the metastasis, differentiation, and drug resistance of cancer (Friedmann-Morvinski and Verma, 2014; Leon et al., 2016; Shibue and Weinberg, 2017). Cancer stem cells can enhance the ability of tumor progression, drug-resistant metastasis, and self-renewal (Beck and Blanpain, 2013). Therefore, we combine analysis of ovarian cancer and mRNAsi to obtain important markers.

The results of the UK Collaborative Trial of Ovarian Cancer Screening (UKCTOCS) study do not show an overall survival advantage to use the Risk of Ovarian Cancer Algorithm (ROCA) testing, thus no screening test exists at this time (Skates, 2003; Jacobs et al., 2016; Tayob et al., 2018). To further elucidate the genes associated with ovarian cancer and their role in the risk of ovarian cancer, we seek to identify key high-risk prognostic genes by mRNAsi in cancer samples. The idea of migration algorithm is used to transfer the prognostic information to single-cell data to assist the identification of cell subpopulations. Based on the conclusion of single-cell data, we return the differentially expressed genes obtained by single-cells to a bulk sample for survival analysis to achieve a precisely targeted treatment effect. Our work aims to: 1) Identify key genes associated with both stemness and prognosis; 2) Find the cell populations associated with stemness and poor prognosis; 3) Identify high-risk core genes in cell populations except for key genes.

To our knowledge, this is an innovative analysis that the bulk sample and individual clinical characteristics of ovarian cancer are combined with stem cell characteristics, and the resulting attributes are migrated to HGSOC single-cell data to assist subsequent analysis of single-cell data. In the past, only cancer and cancer stem cells, gene mutations, and tumor microenvironment have been combined for analysis, but no analysis has been performed to transfer the characteristics of BULK RNA-Seq and cancer stem cells to single-cell data (Ye et al., 2020). Thus, the final results are not only more accurate but also have more diagnostic significance.

In our new analytical process, the key genes related to ovarian cancer are identified as LCP2, FCGR3A, COL1A1, COL1A2, MT-CYB, CCT5, and PAPPA, which may have important significance in ovarian cancaer and drug therapy. With the improvement of our understanding in terms of ovarian cancer subtypes’ composition, some histological specific therapeutic drugs can be used to achieve the effect of precision-targeted therapy. Aiming at the high-risk genes related to ovarian cancer is helpful for the diagnosed patients to carry out risk reduction assessment and preventive surgery (Zhang et al., 2018).

## 2 Materials and Methods

Use bulk RNA-Seq sample and single-cell data of ovarian cancer in this paper. Through calculating the mRNAsi of each sample in bulk RNA-Seq sample, and analyzing the relationship between mRNAsi and clinical features. The stemness-related key gene is obtained by WGCNA analysis. Each cell subpopulation is obtained by analyzing single-cell data, and combined with stemness-related key gene analysis, the target cell subpopulations are obtained.

**FIGURE 1:**
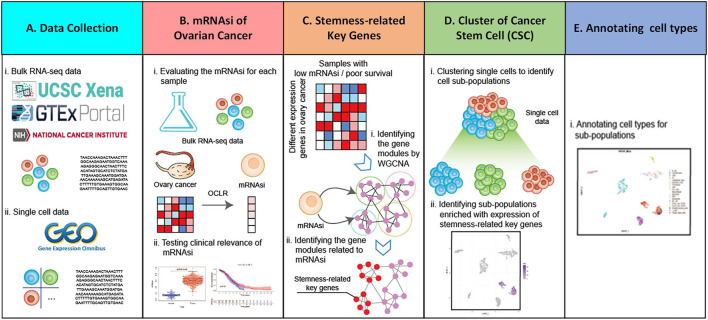
Holistic approach. **(A)** Data collection. **(B)** mRNAsi of ovarian cancer bulk RNA-Seq sample is calculated and analyzed. **(C)** Access to stemness-related key genes. **(D)** Single-cell data of ovarian cancer are analyzed using Seurat and SingleR package. **(E)** Annotation cell types for sub-population.

In the data collection part (Figure 1A), bulk RNA-Seq sample and single-cell RNA-Seq data are collected, among which bulk RNA-Seq sample came from UCSC Xena (https://xena.ucsc.edu/) database and single-cell RNA-Seq data from GEO (https://www.ncbi.nlm.nih.gov/gds) database. After collecting the data, the mRNAsi is analyzed (Figure 1B). The mRNAsi of each ovarian cancer sample data using One Class Linear Regression (OCLR) (Malta et al., 2018) algorithm and combined with bulk RNA-Seq sample is evaluated. Furthermore, the relationship between ovarian cancer mRNAsi and each clinical feature is analyzed, obtaining samples associated with stemness and low survival. In order to obtain the stemness-related key genes (Figure 1C), bulk RNA-Seq of ovarian cancer is analyzed first. The data is used for differential analysis and up-regulated differentially expressed genes are obtained (Ye et al., 2020). Then, low-survival samples and up-regulated differentially expressed genes are used for WGCNA (Langfelder and Horvath, 2008) analysis to obtain stemness-related key genes, and stemness-related key genes are analyzed for enrichment analysis and protein interaction analysis. Meanwhile, using Seurat (Butler et al., 2018) and SingleR (Aran et al., 2019) R packages for cell population cluster of single-cell data from ovarian cancer (GSM5276940, GSM5276943) (Figure 1D). Combining with the distribution of stemness-related key genes in each cell population cluster, the target cell population is determined. The differentially expressed genes between the target cell population and all other cell populations are analyzed to obtain the core genes most related to stemness key genes. After the target cell population is determined, each cell type was annotated using a SingleR (Figure 1E).

### 2.1 Data Source and Preprocessing

The data are mainly from public databases TCGA, UCSC Xena and GEO (Figure 1A). The gene expression RNA-Seq (HTSeq-TPM) data are downloaded from the UCSC Xena database, including 88 normal samples, and 416 tumor samples. Clinical characteristic data of ovarian cancer are downloaded from the TCGA database, including gender (female), age (26–89 years), tumor stage (stageⅠ, stageⅡ, stageⅢ, and stageⅣ), tumor grade (G1, G2, G3, and G4) and patient survival information (survival time and survival status).

The single-cell RNA-Seq data are retrieved from GEO (GSE173682). The data mainly includes four subtypes of endometrial cancer, high-grade serous ovarian cancer carcinosarcoma, and metastasis-associated ovarian cancer (Figure 1A). In the data GSE173682 we used, only 11 scRNA_Seq samples are included, and only GSM5276940 and GSM5276943 are primary ovarian cancer. GSM5276933 GSM5276934 GSM5276935 GSM5276936 GSM5276937 is Endometrium, although the main site of GSM5276939 is Ovary, they are not ovarian cancer in the traditional sense. GSM5276938 and GSM5276942 are metastatic cancers. GSM5276940 and GSM5276943 are carcinoma *in situ*. Here, GSM5276940 mainly contains 8,181 single-cell samples, involving one patient. GSM5276940 mainly contains 6,939 single-cell samples, involving one patient.

### 2.2 Analyze Ovarian Cancer mRNAsi

OCLR method is used to calculate the mRNAsi of each sample in combination with bulk RNA-Seq sample in UCSC Xena. The relationship between mRNAsi and various clinical features is analyzed to obtain the stemness-related low survival samples, which are used in obtaining the stemness-related key genes in Get *Stemness-Related Key Genes*.

#### 2.2.1 Calculate mRNAsi of Ovarian Cancer

For the mRNAsi of ovarian cancer samples, using the One Class Linear Regression (OCLR) algorithm combined with the human stem cell data provided by Progenitor Cell Biology Consortium (PCBC) (https://www.synapse.org) for training, and then quantifying the mRNAsi of our ovarian cancer samples (Figure 1B). Using mRNAsi (0-1) evaluate the mRNAsi of ovarian cancer cells, and the closer the value is to 1, the stronger stemness of cancer cells is.

#### 2.2.2 Analysis of Clinical Features and mRNAsi

Ovarian cancer samples are divided into normal and tumor groups, ovarian cancer samples are divided into two groups based on median age, and ovarian cancer samples are divided into two groups based on mRNAsi. Differences in each group are analyzed. The main grouping is as follows, the samples are divided into two groups based on the median age of all samples in the clinical data. After ranking the samples according to the mRNAsi, the first 1/3 and the last 2/3 are defined as high mRNAsi and low mRNAsi groups. Wilcox’s rank-sum test is used to identify differentially expressed genes (DEGs) in each group. Genes with FDR <0.05 and logFC >1 are identified as differentially expressed genes. To investigate whether cell stemness is associated with patient survival, we use cox regression to test survival differences between the high and low mRNAsi groups. The *p*-values < 0.05 are considered as significant. The sample is defined as stemness-related low survival rates samples, which will be used in the construction of the co-expression network in WGCNA (Langfelder and Horvath, 2008).

### 2.3 Get Stemness-Related Key Genes

The up-regulated differentially expressed genes are obtained by differential analysis using WGCNA. The up-regulated differentially expressed genes and stemness-related low survival samples are analyzed to obtain the stemness-related key genes. The stemness-related key genes are analyzed by enrichment analysis and protein interaction analysis. Stemness-related key genes will be used to identify target cell populations in *Identify Cell Populations of Stemness-Related Key Genes*.

#### 2.3.1 Screening of Differentially Expressed Genes

First, genes with an average expression value of less than 0.2 in all samples are defined as unexpressed and filtered. Then, all samples are divided into two groups, the normal group and the tumor group. The Wilcoxon test is used to identify differentially expressed genes between the normal sample group and the tumor sample group. Genes with FDR <0.05 and logFC >1 are identified as differentially expressed genes.

#### 2.3.2 WGCNA Co-expression Network Analysis

WGCNA is a multiplex analysis method for clustering similar gene expression patterns (Figure 1C). Key genes associated with cell stemness are identified using WGCNA co-expression network analysis based on stemness-related low survival rates samples and differentially expressed genes. Before the construction and analysis of the co-expression network, the quality control of the data is carried out. The samples with missing values and discrete samples are deleted. Selecting the optimal soft threshold β (β = 6) to construct a weighted co-expression network. In addition, the weighted adjacency matrix is transformed into a topological overlap matrix (TOM) to estimate the connectivity of the network. Then, the hierarchical clustering method is used to construct a clustering tree to determine that the module size is set to 80, and the threshold of similarity module merging is set to 0.35.

#### 2.3.3 Identification of Important Modules and Key Genes

The gene sets under the same co-expression module have high topological overlap similarity, and the co-expression degree of these genes is usually higher. Using two approaches to identify important modules associated with mRNAsi. The similarity between modules refers to the correlation coefficient between module and module (MM) characteristic genes, which is used to describe the degree of correlation between each module. Finally, the correlation between a module and mRNAsi is calculated to identify important clinical modules and define the obtained genes as stemness key genes. Genes with *p* < 0.05 are considered key genes. The key gene is defined as stemness-related key gene.

#### 2.3.4 Functional Enrichment Analysis

This analysis is conducted by the cluster profiler package in R (Yu et al., 2012). The biological functions of key DEGs are determined by Gene Ontology (GO) functional annotation and Kyoto Encyclopedia of Genes and Genomes (KEGG) analysis. Stemness-related key gene are selected for analysis, and FDR< 0.05 is taken as the criteria in this section.

#### 2.3.5 Co-expression Analysis and Protein-Protein Interaction Network Construction

The 11.5 version of STRING (https://www.string-db.org) is chosen to investigate and generate the PPI network among key genes, to evaluate the protein-protein interaction (PPI) among key genes. And the key genes are from the stemness-related key genes.

### 2.4 Identify Cell Populations of Stemness-Related Key Genes

The scRNA-Seq (GSM5276940, GSM5276943) of 10X Genomics scRNA-Seq one patient tumor tissue of HGSOC is analyzed (Figure 1D). To identify clusters and find biomarkers for each cluster, selecting Seurat (Butler et al., 2018) and SingleR (Aran et al., 2019) for single-cell data analysis. Single-cell gene expression matrices are entered into R and processed by Seurat 4.0.5 version using principal component analysis (PCA) to reduce the dimension of the data. Elbow plot is used to select the top PCs, which are used downstream for Louvain clustering and visualization using t-distributed stochastic neighbor embedding (tSNE) and uniform manifold approximation and projection (UMAP). Reference-based single-cell RNA-Seq Annotation tool SingleR using HPCA (Human Primary Cell Atlas) reference data to extensively identify cell types of cell populations, through machine learning. Once the cell type is determined, the distribution of each gene in each cell subpopulation is observed in combination with the stemness-related key gene, and the main cell subpopulation is identified as our target cell subpopulation. The marker genes of the target subpopulation are expressed by differential gene expression (logFC >0.5), target differentially expressed genes are identified and survival analysis is performed to find genes that are more closely related to the prognosis of ovarian cancer.

The target cell subpopulations are analyzed and the co-expression network of differentially expressed genes is constructed. Then the genes with a strong correlation (cor >0.6, *p* < 0.05) with stemness-related key genes are found to be identified as a key target gene for the treatment of ovarian cancer.

## 3 Results

### 3.1 Correlation Between mRNAsi and Clinical Features in Ovarian Cancer

**FIGURE 2:**
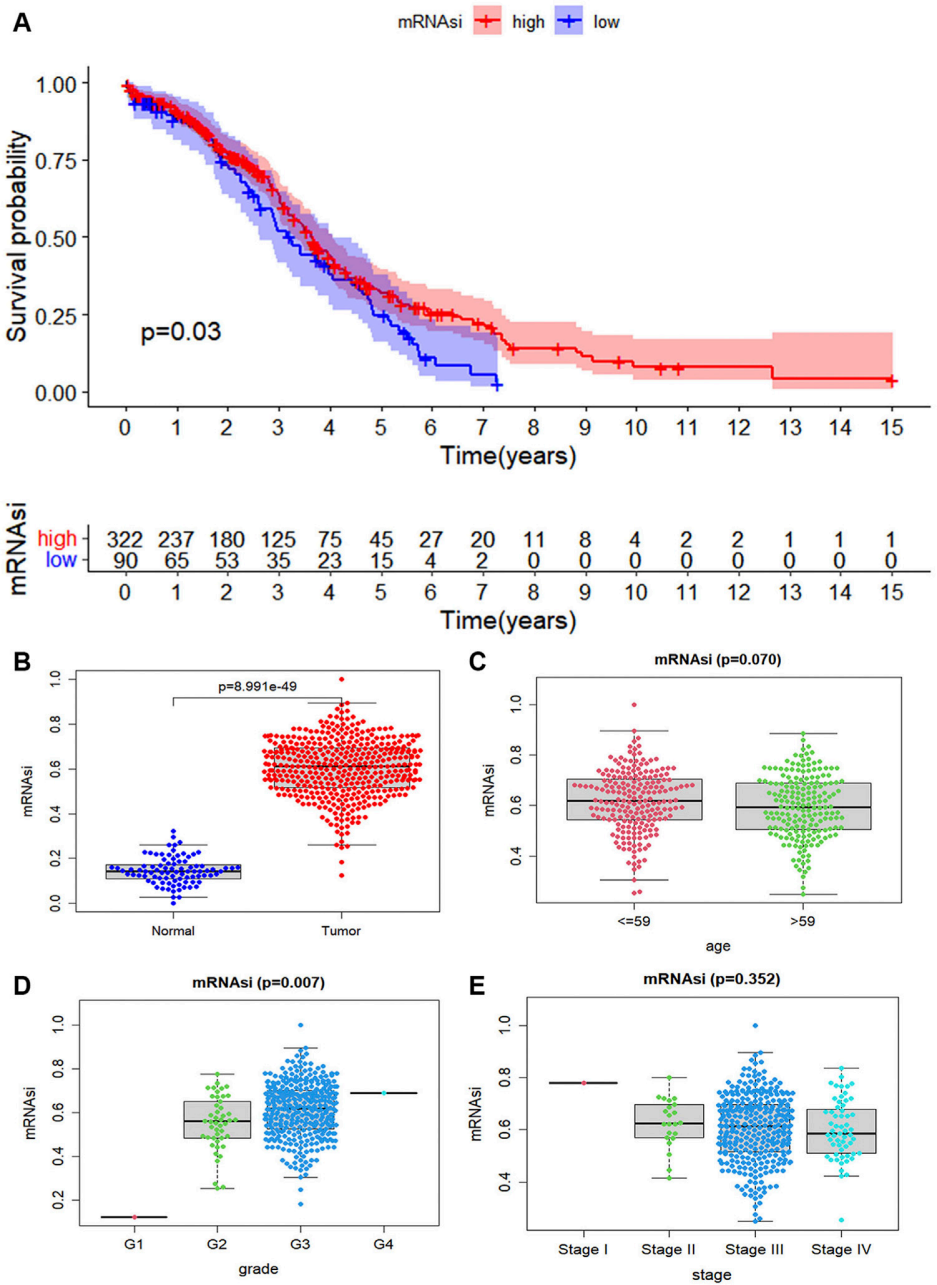
The correlation of mRNAsi index with ovarian cancer. **(A)** The scatter plot shows that the mRNAsi expression in 416 tumor cases is higher than that in 88 normal cases (*p*-value < 0.05). **(B)** The tumor case is divided into two groups based on their mRNAsi score. The Kaplan Meier survival curve shows that the low group enjoyed a lower survival probability. And it is significant statistical differences as a whole (*p*-value < 0.05). **(C)** The tumor cases are divided into two groups based on their age (median age = 59), it is no statistical differences (*p* = 0.07). **(D)** The distribution of the mRNAsi for the clinical grade. The mRNAsi scores increase in more advanced clinical grades, and extremely so in G4 (*p* = 0.07). **(E)** The distribution of mRNAsi scores for stage of ovarian cancer cases.

Considering whether mRNAsi is associated with the clinical features. The tumor samples into two groups according to mRNAsi are divided. There represents a significant difference in survival rate between the high mRNAsi group and the low mRNAsi group, and the Kaplan Meier survival curve shows that the low group enjoyed a lower survival probability (*p* = 0.03, Figure 2A). We explore the characteristic of mRNAsi in ovary cancer and then compare the expression of mRNAsi between cancer and normal samples. The mRNAsi expression in cancer samples is significantly higher than that in normal samples (Figure 2B). Ovarian cancer samples are divided into two groups based on median age. According to the mRNAsi of each sample, there is a correlation between age and ovarian cancer (*p* = 0.07, Figure 2C). According to each grade of ovarian cancer, it finds that the higher the grade of ovarian cancer, the higher the mRNAsi of ovarian cancer samples, which is also due to the discovery of ovarian cancer in the advanced stage (*p* = 0.007, Figure 2D). From the stage of ovarian cancer, it obviously discoveries that there is a big difference between each stage, especially between stage I and stage II, stage III, and stage IV. However, there is no significant difference on the whole, which may be caused by the fact that most ovarian cancer is found in the advanced stage (*p* = 0.352, Figure 2E). Therefore, we can find a clear relationship between the mRNAsi and clinical features of ovarian cancer.

### 3.2 Analyze Stemness-Related Key Genes

#### 3.2.1 Identification of Stemness-Related Key Genes in Ovarian Cancer Based on Weighted Co-expression Matrix Network

**FIGURE 3:**
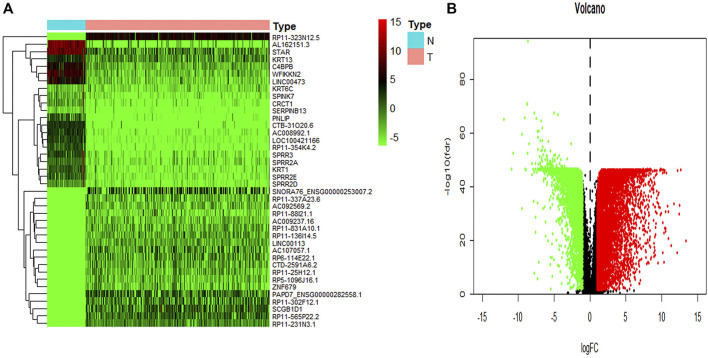
The summary of differential expression genes in ovary cancer samples vs. controls. **(A)** The heatmap shows the top 50 differentially expressed genes. **(B)** The volcano shows a log-fold change of expression of each gene. The red dots represent the up-regulated genes and the green ones represent the down-regulated genes. The black dots represent the gene without significant differential expression in cancer vs. controls.

**FIGURE 4:**
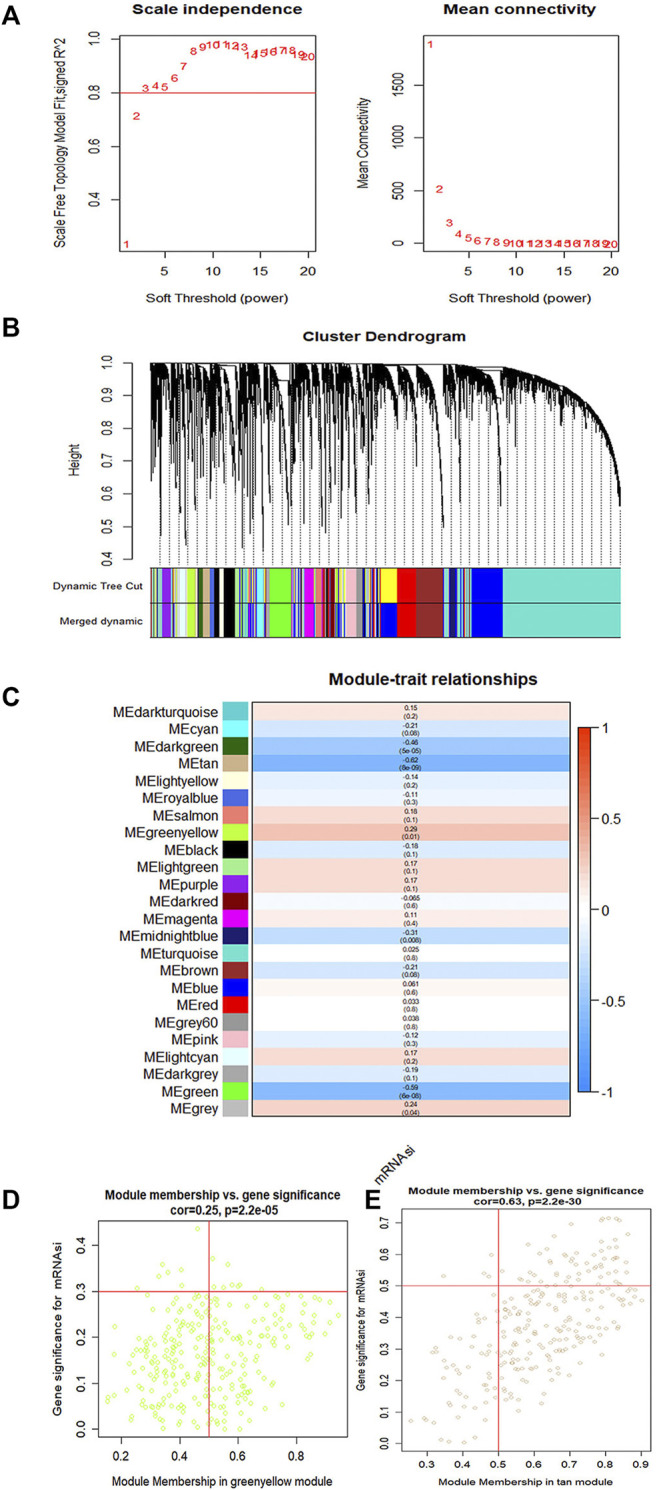
The gene modules are identified by weighted gene co-expression network analysis (WGCNA) and related to the mRNAsi in ovary cancer. **(A)** The indexes are used to determine the power of weight in the co-expression network. **(B)** The branches of the cluster for the different gene modules. **(C)** The correlation between the gene modules and the mRNAsi. **(D,E)** Scatter plot showing the filter of key genes. Each scatter represents a gene. The gene correlated with a module (module membership) and mRNAsi together is considered as the key gene.

To identify key genes related to ovarian cancer more specifically, 12,438 differentially expressed genes have been screened from the normal and tumor tissues of ovarian cancer, including 5,885 up-regulated genes and 6,553 down-regulated genes (Figures 3A,B). Based on samples with a low survival rate and differentially expressed genes, a co-expression network is constructed using WGCNA. To ensure that the co-expression network meets the requirement of a scale-free network, the soft threshold β (β = 6) and the scale-free scale (scale = 0.85) are selected to obtain good average connectivity as shown in (Figure 4A). Modules can be combined by determining the minimum gene number and similarity degree in modules (Figure 4B). A total of 12,438 differentially expressed genes are clustered into 24 co-expression modules (Figure 4C). Among the identified gene co-expression modules, tan and yellow-green co-expression modules are the most closely related to mRNAsi (Figure 4D). There are 171 key genes in the two modules, which are defined as stemness-related key genes for subsequent analysis.

#### 3.2.2 The Cellular Functions and Pathway Analysis of Stemness-Related Key Genes

There are 40 differentially up-regulated genes among the 171 stemness-related key genes. Performing cellular functions and pathway analysis on these up-regulated genes and finding that most of the pathways are related to tumor metastasis and epithelial mesenchymal cells (EMT) (Table 1). EMT, which occurs during tumor progression is highly deregulated, making solid tumors more malignant and increasing their invasiveness and metastatic activity (Ribatti et al., 2020). This is associated with ovarian cancer being found in advanced stages.

**TABLE 1:** Results of stemness-related key gene enrichment analysis.

Pathway	*p*_value
Extracellular matrix organization	1.06E-09
Cell-substrate adhesion	3.73E-06
Wound healing	3.78E-06
Collagen fibril organization	6.48E-06
Collagen catabolic process	1.53E-05
Positive regulation of epithelial cell migration	5.69E-05
Epithelial cell migration	3.79E-04
Cell-cell signaling by Wnt	5.67E-04
Maintenance of location	8.43E-04

#### 3.2.3 Correlation Between Stemness-Related Key Genes at Transcription and Protein Levels

**FIGURE 5:**
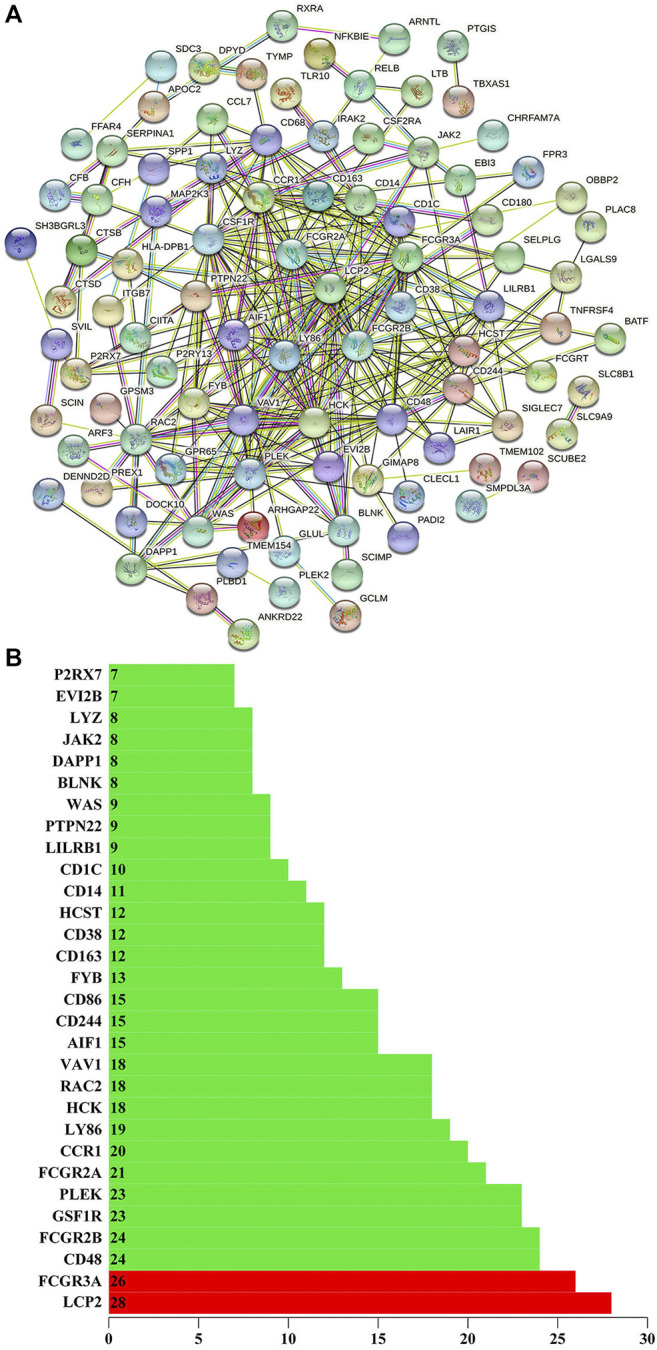
**(A)** Using the STRING (https://string-db.org/) to build the protein interaction network of key genes. **(B)** The number of edges of key genes through the protein interaction network. The *X*-axis represents the total number of edges connected by genes, and the *Y*-axis represents the gene name.

We use the STRING to build the PPI network of the stemness-related key genes (Figure 5A). The more edges the gene connects, the more important the gene is in the PPI network. LCP2 has 28 sides and FCGR3A has 26 sides, so LCP2 and FCGR3A are the most critical proteins (Guo et al., 2020; Ye et al., 2020). Indicating that they are closely related to cancer (Figure 5B). LCP2 can be used as a prognostic biomarker and therapeutic target in anti-tumor immunity (Wang and Peng, 2021). FCGR3A is related to treatment and metastasis in colorectal cancer (CRC) (Zhang et al., 2007).

### 3.3 Target Cell Subpopulations are Identified According to Stemness-Related Key Genes

**FIGURE 6:**
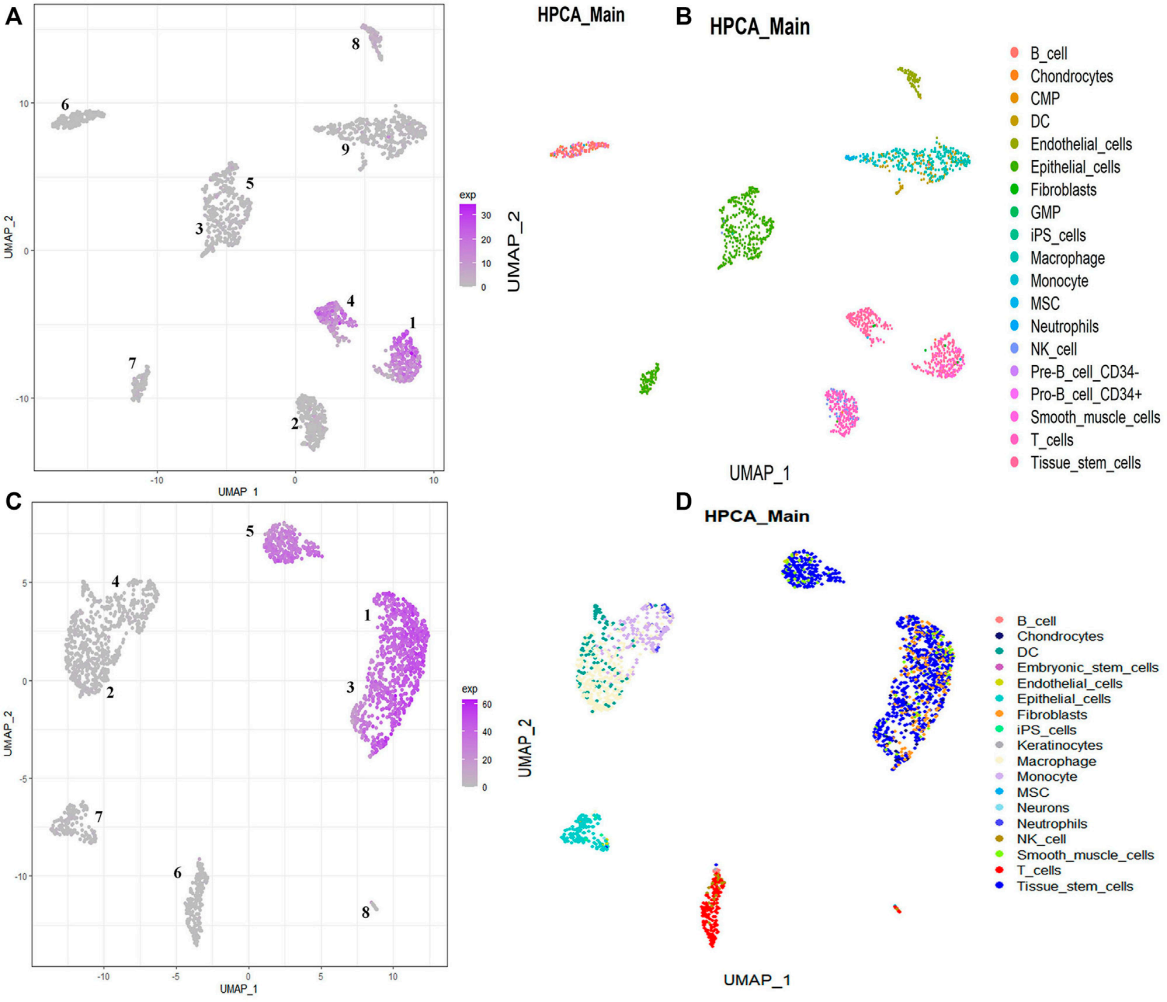
Visualizing each cell population using UMAP. The distribution of individual cell populations in single-cell data. **(A,C)** The main distribution of stemness-related key genes in all cell populations. **(B,D)** The proportion of each cell type in each cell population.

According to the dominant proportion of stemness-related key genes in each cell subpopulation, the key target cell subpopulation can be obtained. By analyzing the single-cell data of GSM5276940, 9 cell populations can be gotten. Based on the proportion of stemness-related key genes in these 9 cell populations, we define the cell population with the largest proportion as the target cell subpopulation, namely cell population 1 and cell population 4 (Figure 6A). By annotating the cells in each cell population, the cell types contained in each cell population can be gotten (Figure 6B). In population 1, there are 291 cells of 7 cell types, including 188 cancer stem cells. Only 60% of cancer stem cells in population 1 could not be defined as the target cell subpopulation. In cell population 4, there were 171 cells in 4 types of cells, among which 161 were cancer stem cells, and 95% are cancer stem cells in the cell population. So population 4 as the cancer stem cell subpopulation is our target cell subpopulation.

By analyzing the single-cell data of GSM5276943, 8 cell populations can be gotten. Based on the proportion of stemness-related key genes in these 8 cell populations, we define the cell population with the largest proportion as the target cell subpopulation, namely cell population 1, cell population 3, and cell population 5 (Figure 6C). By annotating the cells in each cell population, the cell types contained in each cell population can be gotten (Figure 6D). In population 1, there are 562 cells of 4 cell types, including 354 cancer stem cells. Only 69% of cancer stem cells in population 1 could not be defined as the target cell subpopulation. In population 3, there are 331 cells of 4 cell types, including 221 cancer stem cells. Only 68% of cancer stem cells in population 3 could not be defined as the target cell subpopulation. In cell population 5, there were 218 cells in 4 types of cells, among which 257 are cancer stem cells, and 85% are cancer stem cells in the cell population. So population 5 as the cancer stem cell subpopulation is our target cell subpopulation.

Therefore, through the analysis method of this paper. First, stemness-related key genes are identified by bulk RNA-Seq samples combined with the mRNAsi of each sample. Then Cell populations are identified from single-cell data. Finally, Combined with the distribution of stemness-related key genes in each cell subpopulation. This can identify cancer stem cells.

Analyzing the differences between tumor and other cell populations by cell population. We define log2FC > 0.5 as up-regulated gene and log2FC < −0.5 as down-regulated gene. Using the expression data of single-cells with differentially expressed genes and calculating the correlation coefficients between each differential gene according to the spearman correlation. We select stemness-related key genes between genes with cor greater than 0.5 as high-risk stemness-related key genes. A total of 5 stemness-related high-risk genes are obtained (Table 2). Among them, COL1A1, COL1A2, MT-CYB, CCT5, and PAPPA have been reported to be closely related to cancer. Therefore, COL1A1, COL1A2, MT-CYB, CCT5, and PAPPA may be potential genetic biomarkers for the treatment of ovarian cancer.

**TABLE 2:** Stemness-related high-risk genes obtained from the tumor cell population.

Cell population	Reported(cor)
Tissue stem cells population	COL1A2(0.55) Yang et al. (2018)
	COL1A1(0.53) Yang et al. (2018)
	MT-CYB(0.54) Feng et al. (2021)
	CCT5(0.50) Engqvist et al. (2020)
	PAPPA(0.50) Conover and Oxvig (2018)

Each gene is followed by a correlation coefficient(cor) with the stemness-related key gene.

We identified stem cell-related pathways and analyzed LCP2, FCGR3A, COL1A1, COL1A2, MT-CYB, CCT5, and PAPPA with genes in each pathway. As long as more than 50% of the genes in each pathway are associated with LCP2, FCGR3A, COL1A1, COL1A2, MT-CYB, CCT5, and PAPPA *p*_value <0.05, we expect this pathway to be affected. There are altogether 8 such pathways (Table 3).

**TABLE 3:** Stemness-related high-risk genes affect stem cell-related pathways.

Stem cell-related pathways
BEIER_GLIOMA_STEM_CELL_UP
GAL_LEUKEMIC_STEM_CELL_UP
PECE_MAMMARY_STEM_CELL_UP
PECE_MAMMARY_STEM_CELL_DN
HOEBEKE_LYMPHOID_STEM_CELL_UP
GO_HEMATOPOIETIC_STEM_CELL_HOMEOSTASIS
GO_NEGATIVE_REGULATION_OF_STEM_CELL_DIFFERENTIATION
GO_POSITIVE_REGULATION_OF_HEMATOPOIETIC_STEM_CELL_PROLIFERATION

## 4 Discussion

Through our new analysis method ovarian cancer and stem cell characteristics combined with single-cell data analysis, our results are closely related to the development and metastasis of ovarian cancer, and demonstrate the characteristics of cancer stem cells. Cancer stem cells are self-renewing, multipotent properties, and proliferative, giving certain cell subpopulations the ability to initiate, develop, and progress cancer (Matulonis et al., 2016). Different mechanisms contribute to intratumor heterogeneity, including genetic mutations, the microenvironment, and the existence of subpopulations of cancer cells with increased renewal capacity and the ability to recapitulate the heterogeneity found in primary tumors (Kim et al., 2016). Common CSC identification markers include ALDH1A1, CD34, CD24, CD44, CD123, CD133, CD117, and EPCAM (Jin et al., 2009; Kim et al., 2016). These specific CSC markers can be selectively targeted and used to treat invasive, metastatic, and relapse tumors. For example, targeting the overexpressed CD123 marker on CD34^+^ CD38^−^ leukemic stem cells in acute myelogenous leukemia impairs leukemic stem cells homing to the bone marrow and induces a decrease in the overall AML cell repopulation (Jin et al., 2009). Inhibition of developmental signaling pathways that are crucial for stem and progenitor cell homeostasis and function, such as the Notch, Wnt, Hedgehog, and Hippo signaling cascades, and continues to be pursued across multiple cancer types as a strategy for targeting the CSCs hypothesized to drive cancer progression with some success in certain malignancies (Clara et al., 2020). Due to their plasticity and given that CSCs need to be eradicated to prevent malignancy and metastasis, targeting specific niche components relevant to that particular cancer type in addition to standard cancer therapy that tackles the bulk of the tumor bears therapeutic promise (Plaks et al., 2015). High-grade serous ovarian cancer (HGSOC) is the most common pathological type of ovarian cancer and is typically very responsive to platinum-based chemotherapy (Matulonis et al., 2016). Immune therapies have had limited efficacy in high-grade serous ovarian cancer (HGSOC), as the cellular targets and mechanisms of action of these agents in HGSOC are unknown (Wan et al., 2020).

Using our new analytical method, we first examine ovarian cancer, both normal and tumor samples, a total of 12,439 differentially expressed genes are identified in normal, and tumor tissue samples. The clinically relevant survival time and survival status are analyzed together with mRNAsi, and 90 samples of stemness-related ovarian cancer patients with low survival rates are obtained. For these samples, WGCNA further identifies the most significant correlation two gene co-expression modules and mRNAsi. After GO enrichment analysis of 171 stemness-related key genes in the two modules, these genes are primarily involved in the metastasis of ovarian cancer. In addition, in cancer, EMT is associated with the occurrence of tumor invasion, metastasis, and treatment resistance (Pastushenko and Blanpain, 2019). Wnt signal transduction and the role of Wnt-regulated stem cells in the homeostasis regeneration of intestinal, gastric, cutaneous, and hepatocellular cancer environment (Tan and Barker, 2018).

In addition, we analyze the single-cell data from a patient with HGSOC on a 10X data platform. Single-cell data are homogenized and standardized using the Seurat R package, and highly variable genes are selected. After PCA dimensionality reduction, the samples are clustered. And UMAP algorithm is used to visually of the sample. Each cell type is annotated using the SingleR R package. Finally, the main proportion of 171 key genes in each cell subpopulation is obtained by analyzing the bulk sample. Consistent with our preconceive hypothesis, we obtain a key target cell subpopulation.

Through the analysis of stemness-related key genes and tumor stem cell subpopulations, LCP2, FCGR3A, COL1A1, COL1A2, MT-CYB, CCT5, and PAPPA are closely related to ovarian cancer. We consult these genes and find that they are associated with ovarian cancer in different reports. LCP2 is associated with hsa-miR-142 expression in ovarian cancer, and hsa-miR-142-related signaling may lead to progressive loss of cell-cell adhesion (Andreopoulos and Anastassiou, 2012). In the treatment of ovarian cancer, the high affinity and low affinity receptor types of FCGR3A may contribute to clinical outcomes in ovarian cancer treatment (Wang et al., 2017, 125). COL1A1 and COL1A2 may be involved in the occurrence and metastasis of ovarian cancer (Yang et al., 2018). MT-CYB is closely related to oocyte repair and can be cited as a potential target for the treatment of premature ovarian insufficiency (Feng et al., 2021). CCT5 can be used as a prognostic marker of ovarian cancer and can improve the prognosis of ovarian cancer (Engqvist et al., 2020). PAPPA is associated with the growth, invasion and metastasis of ovarian cancer (Conover and Oxvig, 2018). Therefore, these genes can serve as important markers for the treatment and prediction of ovarian cancer.

## 5 Conclusion

In this study, our new method combines bulk RNA-Seq sample with ovarian cancer stem cells, and transfers the obtained attributes to HGSOC single-cell data. Thus a better effect is achieved. And the key genes related to poor prognosis and stem cells are identified through bulk samples. We can obtain the genes that are super closely related to ovary are LCP2, FCGR3A, COL1A1, COL1A2, MT-CYB, CCT5, and PAPPA. The above genes can be targeted for the study of inhibitory agents, so as to achieve the precisely targeted therapy and specific markers of ovarian cancer stem cell populations. At the same time, accurate consultation on risk reduction and preventive surgery is also helpful.

## Data Availability

The Source code and datasets for this work can be obtained from https://github.com/WangZH19/OV_And_Stemness.
